# The Mechanics of Forming Ideal Polymer–Solvent Combinations for Open-Loop Chemical Recycling of Solvents and Plastics

**DOI:** 10.3390/polym14010112

**Published:** 2021-12-29

**Authors:** Ioannis Tsampanakis, Alvin Orbaek White

**Affiliations:** 1 Energy Safety Research Institute, Swansea University, Bay Campus, Swansea SA1 8EN, UK; 958003@Swansea.ac.uk; 2 Chemical Engineering, Faculty of Science and Engineering, Swansea University, Bay Campus, Swansea SA1 8EN, UK

**Keywords:** plastic waste, chemical recycling, mathematical modelling, carbon feedstock, circular economy, open-loop recycling, acrylonitrile butadiene styrene, polystyrene, toluene

## Abstract

The inherent value and use of hydrocarbons from waste plastics and solvents can be extended through open-loop chemical recycling, as this process converts plastic to a range of non-plastic materials. This process is enhanced by first creating plastic–solvent combinations from multiple sources, which then are streamlined through a single process stream. We report on the relevant mechanics for streamlining industrially relevant polymers such as polystyrene (PS), polypropylene (PP), high-density polyethylene (HDPE), and acrylonitrile butadiene styrene (ABS) into chemical slurries mixed with various organic solvents such as toluene, xylene, and cyclohexane. The miscibility of the polymer feedstock within the solvent was evaluated using the Relative Energy Difference method, and the dissolution process was evaluated using the “Molecular theories in a continuum framework” model. These models were used to design a batch process yielding 1 tonne/h slurry by setting appropriate assumptions including constant viscosity of solvents, disentanglement-controlled dissolution mechanism, and linear increase in the dissolved polymer’s mass fraction over time. Solvent selection was found to be the most critical parameter for the dissolution process. The characteristics of the ideal solvent are high affinity to the desired polymer and low viscosity. This work serves as a universal technical guideline for the open-loop chemical recycling of plastics, avoiding the growth of waste plastic by utilising them as a carbon feedstock towards a circular economy framework.

## 1. Introduction

Plastics are undoubtedly one of the most useful materials to mankind. Their versatility can meet almost any requirement, which is something that can be proven by simply taking a glance at their daily use [1]. Despite their benefits, the majority are thrown away after only a single use. More than half of the plastics produced since the 1950s have either ended up in landfill or are now lost to the environment [2]. Currently, the problem is that landfills are swarmed with plastics, whether they are recyclable or not, which results in a huge loss of hydrocarbon that could be utilised otherwise [3]. Recycling plastics via closed-loop recycling renders plastics that are inherently less desirable than virgin plastics, which is mainly due to inferior chemical and physical properties, but they are nonetheless a good source of hydrocarbon material. Hydrocarbons are highly versatile and can be used as feedstock for creating various materials other than plastics; ideally, they would be used to make materials with high asset value and intrinsic beneficial material properties, and this can be done using open-loop recycling. Open-loop recycling is required to make any headway on solving the growing plastic waste crisis so that plastic hydrocarbon can be made available to make other materials rather than being made into more plastics.

The recycling of plastics can be categorised into two methods, mechanical and chemical. Although mechanical recycling is often the first-choice method industrially, there are enough drawbacks to consider an alternative option. In the mechanical recycling process, plastics enter and are converted to recycled plastic (plastic in = plastics out). The process efficiency can vary depending on the mixture of plastics [3]. For example, mechanically recycling different polymers or contaminated polymers result in altered mechanical properties of the end-product, which is usually undesired [4]. Moreover, polymers degrade after undergoing a recycling cycle, leading to the inevitable: plastics ending up in landfills [4]. Therefore, mechanical recycling does not provide a sustainable solution to the growing plastic pollution problem. However, chemical recycling process uses plastics as a hydrocarbon feedstock to make other material as output. Therefore, this process can handle mixtures of plastics, and the polymers can go through further subsequent cycles, therefore proving a more sustainable alternative [4]. Moreover, the chemical recycling process gives opportunity to create a large swathe of material output that can be tailored according to user needs. A full review of chemical recycling techniques, as well as their applications, benefits, and drawbacks is beyond the scope of this work but can be found in the review made by Ragaert et al. [4]. One common trait of these processes is the use of pyrolysis to treat plastics.

The driving principle of chemical recycling is to treat the subject material as a hydrocarbon feedstock for subsequent chemical reactions and processing. Processing is made easier by the use of smaller hydrocarbon molecular units, which are then re-assembled into an open-loop pathway to make newly formed products. Therefore, a first step towards open-loop recycling is the preparation of small molecular hydrocarbon from plastics. Therefore, plastics that are not abandoned in landfill or incinerated are typically treated by pyrolysis. In the conventional pyrolysis process, waste plastics are heated to 500 °C in the absence of oxygen, breaking down the macrostructure of the polymer (plastic) to smaller molecular units [4]. The energy requirements of pyrolysis can be improved by using smaller molecular units at the process entry, and this can be achieved by first dissolving the plastics in appropriate solvents in advance of pyrolysis. Polymers are first dissolved in solvent, and the process is optimised to increase polymer mass fraction [5] and to scission the polymers in the liquid matrix, which is carried out by making polymer–solvent combinations (PSCs). These PSCs are hydrocarbon rich, thus providing the required carbon feedstock for the formation of new materials of high asset value [4,6].

Some of the most challenging plastics include (but are not limited to) polystyrene (PS), polypropylene (PP), high-density polyethylene (HDPE), and acrylonitrile butadiene styrene (ABS), particularly when considered as part of a mixed waste stream (commonly found in practice). Each of these plastics are commonly used in building and construction industries, along with textiles, consumer products, electrical and the electronics industry. Moreover, in the case of ABS, there is increased demand due to the growth of additive manufacturing industries. Therefore, the aim of this study was to elucidate a universal approach for recycling these plastics. This was done by choosing appropriate mathematical models and by optimising the process using various engineering parameters. The results could be used to predict and influence the design of engineering operational units towards the large-scale chemical recycling of plastics.

## 2. Physical and Chemical Context

### 2.1. Solubility of Plastics and Basic Polymer Solution Thermodynamics

The selection of an appropriate solvent is critical for the dissolution of polymers and plastics in particular. The chemical structure of a polymer plays a major role in its solubility to a solvent [5]. For polymer dissolution to occur, the solvent molecules must be separated from each other by the solute. Similar attractions between the molecules of both components achieve efficient dissolution [7]; the principle is known as ‘like dissolves like’ [5]. The dissolution of substances in each other is accomplished if both their intermolecular forces (Van der Waals) are similar and the composite forces are made up in the same way [7]. Solubility parameters or cohesive energy parameters (CEDs) have many applications in industry, including polymer compatibility and dissolution of polymers [5]. They are derived from the energy requirements of vaporisation of a liquid [8].

#### 2.1.1. Hildebrand Solubility Parameters and Thermodynamics of Polymer Dissolution

The term ‘solubility parameter’ was first introduced by Hildebrand and Scott. The Hildebrand solubility parameter, *δ* (MPa1/2), is defined by the following equation:

(1) δ=EV

where *V* is the molar volume of pure solvent and *E* is its energy of vaporisation [8].

The free energy of mixing regulates the dissolution process of an amorphous polymer [9]. The Gibbs free energy change for the solution process is governed by the following relationship [9].

(2) $$\Delta G_{m}=\Delta H_{m}-T\times \Delta S_{m}\;$$

where Δ*G_m_* is the Gibbs free energy of mixing, Δ*H_m_* is the enthalpy change of mixing, Δ*S_m_* is the entropy change in the mixing process, and *T* is the absolute temperature. Thermodynamics requires that the free energy of mixing must be zero or negative for spontaneous dissolution of the polymer [8]; otherwise, two or more phases result from the mixing process [5]. Therefore, the heat of mixing (Δ*H_m_*) must be smaller than the entropic term (*T*Δ*S_m_*) for spontaneous polymer dissolution (Δ*G_m_* ≤ 0) according to Equation (2) [7]. Hildebrand and Scott proposed an equation for the heat of mixing of a binary solution.

(3) $$\Delta H_{m}=\varphi_{1}\varphi_{2}V_{m}{\left(\delta_{1}-\delta_{2}\right)}^{2}\;$$

where *φ*_1_ and *φ*_2_ are the volume fractions of solvent and polymer, respectively, *δ*_1_ and *δ*_2_ are the Hildebrand solubility parameters for solvent and polymer, respectively, while *V_m_* is the volume of the mixture. However, Patterson et al. have shown that the non-combinatorial free energy of solution is given by the right-hand side of the previous equation instead of Δ*H_m_*, even at the condition of using only positive heat of mixing [8]. The new formula proposed is shown below:

(4) $$\Delta G_{m\;\left(noncomb\right)}=\varphi_{1}\varphi_{2}V_{m}{\left(\delta_{1}-\delta_{2}\right)}^{2}\;\;$$

Both positive and negative heats of mixing can be derived from this formula, and its results are consistent with the Prigogine corresponding states theory (CST) of polymer solutions. The difference in solubility parameters for the solvent–solute system is important in determining the system’s solubility. A match in solubilities (*δ*_1_ = *δ*_2_) leads to zero, therefore cancelling the term; this combined with the positive entropy change upon simple mixing ensures that the solution is possible from a thermodynamic perspective [8]. Considering the limiting case, Δ*G_m_* = 0, Equation (2) becomes:

(5) $$\Delta G_{m\left(noncomb\right)}=T\Delta S_{m}.\;$$

From this formula, it is seen that the change in entropy dictates how closely the solubility parameters must match for dissolution to occur [8]. However, the predictions with the Hildebrand solubility parameters are inconsistent due to the absence of some factors that affect solubility. More specifically, they do not account for any specific hydrogen interactions, especially hydrogen bonding, neither for the effects of morphology (crystallinity) nor crosslinking. It has also been found that changes in temperature and concentration cannot be taken into account with the Hildebrand solubility parameters [5]. Therefore, further refinement is required to make a practical understanding possible.

#### 2.1.2. Hansen Solubility Parameters (HSP)

Hansen, using the extensive work done over the years to improvise the Hildebrand solubility parameters, accounted for molecular interactions and developed solubility parameters based on three specific interactions [5]. The most general are the nonpolar interactions, which are derived from atomic forces; they are also referenced as ‘dispersion interactions’ in the literature. Since all molecules are built from atoms, all molecules contain these interactive forces. There are three specific interactions:

1. The cohesive energy from dispersion interactions is described as *E_D_*.
2. The permanent dipole–dipole interactions. These cause a second type of cohesion energy, the polar cohesive energy, *E_P_*. These interaction parameters are also found in most molecules to one extent or another.
3. Hydrogen bonding, *E_H_*, also known as the electron exchange parameter, resembles the hydrogen polar interactions. In a more simplified approach, this third term is used to collect all the energies from the rest of the interactions not included in polar or dipole cohesive energy components. It has been proven that the Hansen hydrogen bonding has served well and is of practical importance.

There are naturally more interactions that occur and can be counted for, but these three mentioned herein are adequate to describe the majority of cased [8]. Together, they contribute to the basic equation that governs the assignment of Hansen parameters as shown below:

(6) $$E=E_{D}+E_{P}+E_{H}\;\;$$

where *E* is the total cohesion energy. Dividing each term from the previous formula by the molar volume gives the square of the total Hildebrand solubility parameter.

(7) $$\frac{E}{V}=\frac{E_{D}}{V}+\frac{E_{P}}{V}+\frac{E_{H}}{V}\;$$

Using Equations (1) and (7), the total Hildebrand parameter is shown to be:

(8) $$\delta^{2}=\delta_{D}{}^{2}+\delta_{P}{}^{2}+\delta_{H}{}^{2}.$$

The calculation of these three parameters is best evaluated from experiments rather than using molecular orbital calculations or calculations based on theoretical approaches [8]. Most theoretical methods to calculate HSP are inconsistent, and while some experimental methods are accurate, they can be time-consuming [5]. Small proposed his theory on determining solubility parameters using a molar attractive constant, F, to overcome the inconsistencies of other methods. The molar attractive constants at 25 °C for various common functional groups are tabulated in the literature. The sum of the constants for each molecule gives the following relationship.

(9) $$E=\frac{{\left(\sum F\right)}^{2}}{V}\;\;$$

This leads to the Hildebrand solubility parameter, *δ*, using Equation (1) [5]. Panayiotou et al. have approached the solubility calculation via another method. They have used a statistical thermodynamics approach to predict all three HSP parameters by using only the molecular structures of the compounds. After comparing the calculated Hildebrand solubility parameter to experimentally measured values, it was proven that the differences between the two are minor; thus, the method is considered fairly accurate [10].

#### 2.1.3. Models for the Prediction of Polymer Solubility

**Figure 1:**
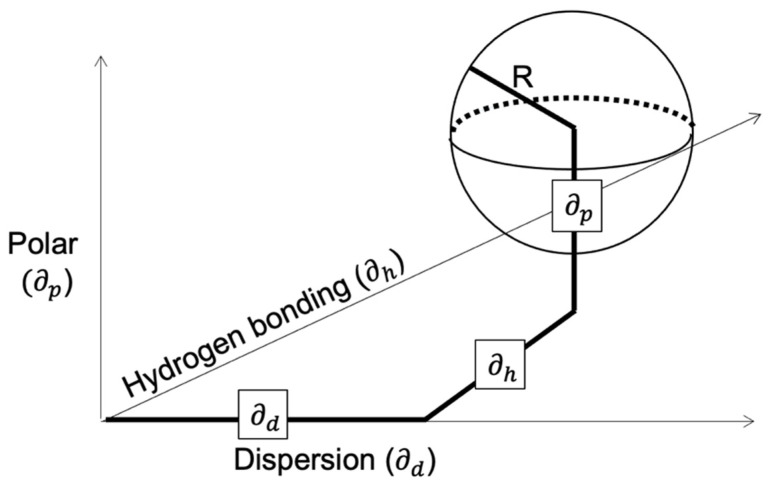
Representation of the Hansen 3D model of a solubility sphere with radius R, as determined by the polar, dispersion, and hydrogen bonding components, respectively.

The solubility parameters mentioned can be used in various techniques to predict the polymer solubility of any solvent. Hansen developed a 3D graphical solubility model, which is characterised by a sphere. The centre of the sphere has the HSP of the polymer, while the radius of the sphere, *R*_0_, is termed the interaction radius. The boundary of the sphere is determined by the requirement that ‘good’ solvents have a distance from the centre, *R*_α_, where *R*_α_ < *R*_0_. Values for the interaction radius are tabulated in the literature for various polymers, whereas the distance from the centre is calculated via the following formula:

(10) $$R_{a}{}^{2}=4{\left(\delta_{D,p}-\delta_{D,s}\right)}^{2}+{\left(\delta_{P,p}-\delta_{P,s}\right)}^{2}+{\left(\delta_{H,p}-\delta_{H,s}\right)}^{2}\;\;$$

where the solubility parameters with the subscript *s* are for the solvent and *p* for the polymer, and the constant ‘4’ is derived from correlations. The proposed sphere is presented in Figure 1.

A single parameter derived from this formula to describe the solubility is the relative energy difference (*RED*) number.

(11) $$RED=\frac{R_{a}}{R_{0}}\;\;\;$$

The *RED* number has three scales. For *RED* = 0, there is no energy difference; thus, complete miscibility is anticipated. An *RED* ≈ 1 describes the boundary condition between *RED* < 1 indicating high affinity, as opposed to *RED* > 1, where the affinity is progressively lower [8]. 

Hansen also developed a method for predicting the solubility of polymers in a mixture of solvents. An interesting discovery that occurred in some cases was that even if both solvents are bad solvents, a good ratio in their mixing can make them very effective in the dissolution of some polymers. The practicality of this approach is characterised by the fact that they released proprietary software to test pure or mixed solvents [11].

Another method to predict polymer solubility was reported by Teas in 1968. Using the HSP from either the literature or experiments, Teas developed some fractional parameters and then used a ternary diagram, where each edge of the triangle represents the corresponding fractional parameter. More specifically, the lower right corner represents the parameter related to the dispersion forces (*f_d_*), the lower left corner represents the hydrogen bonding contribution(*f_h_*), and the top corner represents the polar forces (*f_p_*) [12]. The organic solvents are typically grouped closer to the lower right corner, since all of them have a high degree of dispersion forces [7]. When the solubility of any polymer added to any solvent is tested by either experimental or theoretical methods, a polymer solubility window can be determined and plotted on the Teas graph [7]. The boundaries can be accurately determined by the possibility of dissolution of the polymers tested in mixtures of the liquids in the borders of the solubility window [7]. The solubility window of the ternary diagram can also be used as a rough guide for the effects of solvent blends [7]. The fractional parameters are calculated via the following formulas, and the final formula needs to be true for the calculations to be valid.

(12) $$f_{d}=100\times \left(\frac{\delta_{D}}{\delta_{D}+\delta_{P}+\delta_{H}}\right)\;\;$$

(13) $$f_{p}=100\times \left(\frac{\delta_{P}}{\delta_{D}+\delta_{P}+\delta_{H}}\right)$$

(14) $$f_{h}=100\times \left(\frac{\delta_{H}}{\delta_{D}+\delta_{P}+\delta_{H}}\right)\;\;$$

(15) $$f_{d}+f_{p}+f_{h}=100\;\;$$

#### 2.1.4. Rules of Thumb and Solvent Selection

A chemical will be a good solvent for a specific polymer if one of the following ‘rules of thumb’ are valid, wherein the subscript 1 denotes the solvent polymer and subscript 2 denotes the polymer [8]:

1. Using the Hildebrand solubility parameters.
  (16) $$\left|\delta_{1}-\delta_{2}\right|\leq 1.8{\left(\frac{\mathrm{cal}}{{\mathrm{cm}}^{3}}\right)}^{0.5}\approx \;3.6819\;{\mathrm{MPa}}^{0.5}\;\;\;$$
2. Using Hansen Solubility Parameters (HSP) in the Formula (10) under the condition of *R*_0_ ≥ *R*_α_.
3. Using the Flory–Huggins parameter, χ_12_ ≤ 0.5. The lower the parameter, the greater the miscibility. Values above 0.5 indicate insolvency. The ‘chi’ parameter is estimated from the following set of formulas.
  (17) $$\chi_{12}=\frac{VA_{1,2}}{RT}\;$$
  (18) $$A_{1,2}={\left(\delta_{D2}-\delta_{D1}\right)}^{2}+0.25{\left(\delta_{P2}-\delta_{P1}\right)}^{2}+0.25{\left(\delta_{H2}-\delta_{H1}\right)}^{2}$$
  where *V* is the reference volume, measured in cm^3^/mol, *R* is the gas constant, *T* is the temperature in Kelvin, and *A* has units of MPa.

#### 2.1.5. Temperature Dependence on Solubility Parameters

Although the methods used to determine the solubility parameters by Stefanis and Panayiotou are at 25 °C, it is safe to assume that the values remain constant for the solvents until they evaporate. Therefore, recalculation for higher temperatures is possible but not necessary. Generally, higher temperatures result in an overall increase in the rate of solubility/diffusion/permeation as well as larger solubility parameter spheres. The HSP decreases with increased temperature, as shown in the literature. Therefore, a non-solvent can turn into a solvent, but a boundary solvent can either be a good or a bad solvent with increased temperature. In practical terms, it is safe to assume that solubility parameters remain constant in the absence of any phase change occurs (evaporation, solidification) [8]. 

In this manuscript, the techniques of the Hansen solubility sphere, difference of Hildebrand parameter, and Flory–Huggins parameter were used to predict the solubility of the plastic feedstock in selected solvents. Moreover, a Teas graph with the polymer solubility window of each method was plotted as an additional criterion of comparison between the solubility methods.

### 2.2. Polymer Dissolution Models

Over the years, many polymer dissolution models have been developed—both empirical as well as mathematical. Although empirical models are easier to use due to their heavy reliance on empiricism, mathematical models are attractive due to their mathematical expressions, which correlate multiple parameters and yield significant results. Moreover, mathematical models can be used to develop processes in simulation software, thus abandoning the need for experiments that can be resource intensive. Therefore, a search for the most appropriate dissolution model was conducted and reported herein.

Ueberreiter was one of the pioneers in the field of polymer dissolution [13]. He studied the structure of the surface area of glassy polymers and observed four different layers, which were: (i) liquid boundary layer, (ii) gel layer, (iii) solid swollen layer, and (iv) infiltration layer. He also proposed the terms ‘normal dissolution’ and ‘dissolution by cracking’. The difference lies in the existence or absence of a gel layer; if a gel layer exists during polymer dissolution, then it is termed as ‘normal dissolution’. Moreover, Ueberreiter ran experiments and concluded that the dissolution rate was inversely proportional to the polymer molecular weight. Over time, various mathematical models have been derived to describe the dissolution of polymers in different solvents [14], resulting in five approaches to describe polymer dissolution thus far. Each one was investigated, and the most suitable was chosen. Initially, Fickian equations were used to describe the dissolution of the polymers phenomenologically along with external mass transfer approaches. An effort to describe the model via stress relaxation and molecular theories was also made. One of the latest methods developed was the anomalous transport via scaling laws. However, the most recent and widely used due to its great depth is the development of molecular theories in a continuum framework.

#### 2.2.1. Phenomenological Models

This approach was based on Fickian equations, which were used to describe the solvent penetration into the polymer. Tu and Ouano were the first to use this model in the late 1970s [15]. They studied the dissolution of polymethylmethacrylate (PMMA) and PS in butanone (MEK) by defining a key parameter, R_d_, which is the rate at which the polymer transforms from a gel-like phase to a solution. They claimed that the process was controlled by either diffusion or disassociation, depending on which hindered the dissolution. A multi-phase Stefan problem was created to mathematically describe the associated physics and was solved numerically by using a Crank Nicholson scheme [16]. Experimental results for PS dissolution in MEK were compared to those predicted by the model to verify its validity. However, this early model failed to yield more insight into the dissolution process, since the molecular properties of both the polymer and solvent were not considered when determining the disassociation rate [14].

Devotta et al. developed another phenomenological model in which the dissolution of spherical polymeric particles was examined [17]. A mass transfer rate, *k_d_*, was calculated from Ranz’s correlations, which deals with single spheres, while the Fickian equations were solved numerically using a Crank–Nicholson scheme. One of the aspects investigated was the relation of the particle size to various parameters in a PS/cyclohexane system. The dissolution time was almost constant for particles ranging from a few microns to 50 μm, concluding that the critical particle size was 50 μm. Another observation was that the critical particle size would increase at higher agitation. A comparison between the model’s results and experimental results was also made, and the authors reported good agreements. Even though this approach seemed promising, there were assumptions whose nature was not clear; thus, some parameters could not be obtained experimentally [14]. Overall, despite the inadequate explanation, Devotta’s model was very useful for the engineering community, therefore justifying some of his assumptions. The correlation of the model with experimental data influenced the success of this model.

#### 2.2.2. External Mass Transfer Models, Stress Relaxation, and Molecular Theories

The two models, namely external mass transfer and stress relaxation theories, were both deemed unsuitable to describe the polymer dissolution process due to limitations in each of them. Regarding the external mass transfer model, although it seemed intuitive, experiments have shown that the effects of external mass transfer are insignificant to the dissolution of polymers. Moreover, the external mass transfer models failed to explain the swelling time needed before the dissolution process; therefore, they were discarded [14]. Regarding stress relaxation and molecular theories, although these approaches accounted for both solvent penetration and polymer dissolution, too many assumptions were involved to describe the process. The assumptions required parameters that could only be measured experimentally, which was a major drawback of the model. In addition, there was no effort to compare the results of the stress relaxation models to experimental results, which is something that further repelled this approach [14].

#### 2.2.3. Anomalous Transport Models and Scaling Laws

Peppas et al. proposed a model for the dissolution of polymers based on chain disentanglement [18]. The dissolution of PS and PMMA in MEK both theoretically as well as experimentally was studied, and a general expression for the disentanglement time was derived. Polymers are structures entangled in a confined space, and a specific time is needed for the polymer chains to move from the entangled state to the dissolved state in a liquid. This time was named disentanglement time, *t_d_*. Furthermore, the idea of the dissolution clock was proposed. In this concept, the clock is initially set at zero at each point of the polymer and starts only after a critical solvent concentration is reached. When the clock time becomes equal to the disentanglement time, the polymer at that point dissolves. Even though this method was very significant in describing the polymer dissolution, it failed to take into account the solvent concentration history and the effect of viscoelastic properties of the polymer to the dissolution process [14].

#### 2.2.4. Molecular Theories in a Continuum Framework

In this approach, a model was developed for one-dimensional solvent diffusion in a thin polymer slab of thickness 2 L by Narasimhan and Peppas [19]. During the initial stage of dissolution, a glassy polymer of thickness 2 L starts swelling due to the penetration of the solvent into it, and there is a simultaneous transition from the glassy to the rubbery state. There are two distinct fronts: a swelling interface that moves towards the centre of the slab and a polymer/solvent interface that moves outwards until the concentration of the penetrant in the polymer exceeds a critical concentration; then, it moves inwards. The entire concentration field is split into three regions, the “concentrated” regime, the “semi dilute” regime, and the “dilute” regime. The first one represents the swollen polymer, the second represents the diffusion boundary layer with a constant thickness δ, and the third is the fully dissolved region, where the polymer chains freely move in the solvent. In the first regime, the authors used Fickian equations to describe the diffusion of the solvent in the polymer and derived a solvent flux equation as a sum of contributions from diffusive and osmotic pressure terms, where the osmotic pressure in the solvent flux depends on the viscoelastic properties of the polymer. They observed that the solvent flux is proportional to the gradient of the stress and developed a relationship for the viscosity of the solution. In the boundary layer regime, the layer thickness was estimated and defined the limits of the boundary layer on both sides. The idea of the reptation time was introduced; it is the minimum time required for the polymer to disentangle and move out of the gel. It was noted that there was no polymer disentanglement until the reptation time had elapsed, and after that, the flux was determined to be disentanglement-limited. During disentanglement of the chains, the polymer concentration in the boundary layer increases until it reaches an equilibrium value, u_2,eq__._, and it remains there until the polymer is fully dissolved. By assuming that an equilibrium exists between the chemical potential of the solvent in the swollen polymer and that in the diffusion boundary layer, the authors could obtain the solvent concentration on the gel side of the gel–liquid layer. Finally, they managed to derive the following expressions for the reptation time and the disentanglement rate. A more in-depth explanation of the model is presented elsewhere [19]. The equation for the reptation time is:

(19) $$t_{rep}=\frac{3\pi \eta_{1}}{kT}{\left(\frac{N}{Ne}\right)}^{3.4}\alpha^{3}\;\;\;$$

where *η*_1_ is the solvent viscosity (Pa·s), *k* is Boltzmann’s constant (m^2^·kg·s^−2^·K^−1^), *T* is temperature (K), *N* is the number of monomers in the chain, *Ne* is the number of monomers per entanglement segment, and *α* is the average length of a segment between entanglements. Using the same expressions as those derived by the authors in their model, Equation (19) becomes:

(20) $$t_{rep}=\frac{0.01368\eta_{1}}{T}u_{2}{}^{1.9}N^{3.4}\;\;$$

where *u*_2_ is the polymer volume fraction, and the number 0.01368 is the combination of *k*, *Ne*, and *α*. 

The disentanglement rate was defined as:

(21) $$k_{d}=\frac{4.5985\times 10^{-9}T}{\eta_{1}}u_{2}{}^{-1.9}N^{-2.9}.\;\;$$

After the reptation time has elapsed, the polymer disentangles linearly with time at a rate of *k_d_*.

This approach is the most up to date for dissolving plastics in solvents, so it was used to examine the dissolution of the waste plastic. In some cases, empirical models developed for the dissolution of plastics were also considered to overcome the limitations of the used method. 

## 3. Process Selection

### 3.1. Material Selection

The intended design is to produce 1 tonne/h of a plastic–solvent slurry. Although some research has been conducted regarding the upper limits of the plastic that can be dissolved in a solvent [14], none of the mathematical models has been capable of accurately predicting larger scale applications yet. Therefore, an empirical approach was used to select the appropriate plastic-to-solvent ratio. Nauman et al. reported that the concentration range at which polymers are optimally dissolved lies between 5% and 20% by weight [20]. Therefore, for the current process, a plastic concentration of 5 wt % (*w*/*w*) was assumed. If a higher plastic concentration were to be used, the dissolution time would be longer, and the viscosity of the PSC would be increased due to the increased plastic concentration. A higher viscosity would require more energy input for agitation and pumping of the PSC.

The plastic feedstock chosen was commercial waste plastic. However, since every plastic dissolves differently depending on the solvent, the plastic feedstock was specified and assumed to consist of equal quantities of rigid plastics, 25% PS, 25% PP, 25% HDPE, and 25% ABS, for simplicity. Thus, the material feedstock would be 12.5 kg of each plastic type, which, when mixed to the solvent, would sum the required output of 1 tonne/h. 

Plastics can be dissolved in a variety of pure solvents or blends of them. Solvent selection is a critical parameter for the process, since a good solvent can greatly accelerate the dissolution. The solvent selection was influenced by empirical work [6]. In this process, 21 solvents were chosen, and their ability to dissolve the four types of plastic feedstock was tested. Furthermore, the solubility of the four polymers was tested on eight binary mixtures of the 21 solvents. The combinations were done arbitrarily using the application available from the Hansen website to predict the HSP of a mixture of solvents. Therefore, the solubility of the plastics to the binary solvent combinations was only tested via the Hansen method. The affinity of each plastic in the different solvents and mixtures of solvents varied depending on the method used (Hansen, Hildebrand, Flory–Huggins, and Teas) as seen in Table 1, Table 2 and Table 3.

The HSPs for both the solvents and plastics were obtained from the literature [8] (supplementary file, Table S1). The distance from the centre of the sphere, *R_a_*, was calculated via Equation (10) (supplementary file, Table S2) and then compared to the interaction radius of the polymer, *R*_0_, by the *RED* method. Regarding the mixtures of solvents, *R_a_* was obtained directly from the Hansen website [11]. The results showed that acrylonitrile–butadiene–rubber had higher affinity to more solvents or solvent combinations, {twenty} compared to polystyrene {fifteen}, polypropylene {fourteen}, and high-density polyethylene {six}. However, all four plastics could not be dissolved in any pure solvent, whereas only four pure solvents (benzene, hexadecane, toluene, and xylene) yielded an average *RED* < 1. Furthermore, all four plastics could be simultaneously dissolved in only three binary solvent blends.

For the Hildebrand method calculations, the HSPs of both plastics and solvents were obtained from the literature [8] and used to calculate the total Hildebrand parameter of each component via Equation (8). Then, the Hildebrand parameters of the solvents were compared to those of the plastics via Equation (16). The mixtures of solvents were not tested by this method. The plastics were miscible in most solvents, as indicated by the tabulated results. However, HDPE was miscible in less solvents than PS, PP, and ABS. In total, ten solvents could ideally dissolve all four plastics simultaneously.

The HSPs were also used for the Flory–Huggins method in order to calculate the parameter A_1,2_ via Equation (18) (supplementary file, Table S3). The reference volume, V, of the components was obtained from the literature [8] (supplementary file, Table S3). These parameters were used to calculated χ_1,2_ according to Equation (17). The condition for high affinity was χ_1,2_ ≤ 0.5. The solubility was tested in atmospheric conditions and room temperature. According to this method, only four of the total solvents could dissolve all four plastics. 

**Figure 2:**
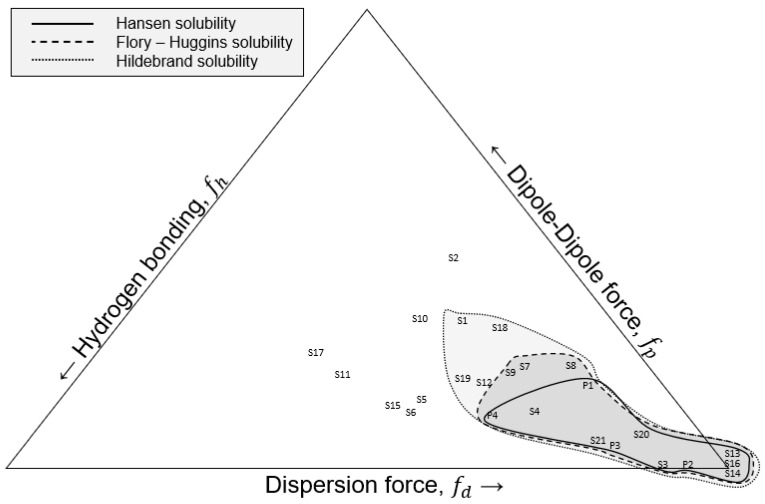
Teas graph of various plastics and solvents according to the hydrogen bonding, dipole–diploe force, and the dispersion force, respectively. Lines show the boundary conditions for three models including the Hansen solubility sphere, the Flory–Huggins solubility, and the Hildebrand solubility.

The HSPs were used in Equations (12)–(15), and the results (supplementary file, Table S4) were used to construct a Teas Graph, as seen in Figure 2 and Table 4 and Table 5. The components were all accumulated toward the lower right side of the graph, as stated in the literature [7]. The legend shows where each solvent or plastic is placed on the graph as well as the polymer solubility windows according to the different solubility identification techniques.

Overall, the miscibility of the plastics in various solvents was tested in three methods, and a visual representation of the polymer solubility window from each method was shown on the Teas plot. The Hildebrand method showed that two-thirds of the solvents (14 out of 21) could be used to dissolve the plastic feedstock compared to nine overall according to the Flory–Huggins method. Only four solvents could dissolve all four plastics simultaneously according to the solubility sphere created from the Hansen Relative Energy Difference method. However, as seen from the Table 1, Table 2 and Table 3, there are some solvents that cannot dissolve all four plastics simultaneously; they could potentially dissolve a very significant portion of the feedstock according to the average dissolution calculations. A comparison between the solvents and non-solvents in the Hildebrand and Flory–Huggins methods reveals that ethyl acetate, hexane, methyl ethyl ketone, and tetrahydrofuran were just above the Flory–Huggins threshold of good solvents, thus making them borderline solvents. However, acetone appeared to be far from that border. A comparison of the solubility results from the Flory–Huggins and Hansen methods revealed a similar result, where chloroform, cyclohexanone, 1,2 dichlorobenzene, dichloromethane, and heptane just fall out of the ‘good’ solvent criteria according to the *RED* method. The same applied when comparing the Hildebrand and Hansen methods. Acetone is again proven to be a non-solvent with all the plastics according to the *RED* method. Furthermore, the results of each method were graphically shown on the Teas plot. The polymer solubility window was shrinking depending on the method with Hildebrand having the widest and Hansen having the smallest window.

An examination of the nature of the organic solvents resulted in the observation that alcohols (methanol, ethanol, hexafluoro-2-propanol, cyclohexanol), phenols (m-cresol), and nitrogen-containing compounds (acetonitrile, dimethylformamide) were generally bad solvents. Ketones (acetone, cyclohexanone, methyl ethyl ketone) and esters (ethyl acetate) were not good solvents, either. On the other hand, aromatic molecules containing only carbons and hydrogens such as benzene, hexadecane, toluene, and xylene are very good solvents. This led to the conclusion that the presence of oxygen or nitrogen in any organic compound as well as its non-aromaticity enhance the probability that the compound is a non-solvent. This was also linked to the polarity of the compounds. The phrase “like dissolves like” is well known. Alcohols, phenols, nitrogen compounds, ketones, and esters are considered polar molecules, whereas symmetric hydrocarbons containing solely carbon and hydrogen are non-polar. In this study, all the plastics used can be identified as non-polar; therefore, non-polar solvents are the better choice. However, as observed by the binary mixtures of solvents created from the Hansen website, the presence of alcohols can enhance the solubility of the mixture. Two notable results are present in Table 1, where cyclohexane is mixed with either cyclohexanol or ethanol. The effects of nitriles to binary mixtures with good solvents also showed promising results. Binary mixtures with ketones, esters, phenols, and amides were not tested. Moreover, we note that in practice, many plastic materials are found to contain additives and/or binders that were not accounted for this in this study. In the case of lacquers and plasticisers, for example, their presence is known to adversely affect dilution by hydrocarbon, as they tend to reduce the tolerance for the presence of hydrocarbons. However, given the broad range of materials covered in Table 5, we anticipate that professionals who are skilled in the art of chemistry and/or chemical engineering would be able to extrapolate from the list of compounds which might be most comparable to their specific binder or plastic filler material. We would hope that should an industry or specific sector use this work, they would be able to quickly determine similarities for their specific compound from within the broad range of compounds that we have included in Table 5.

The solvents that were ultimately selected for this process were toluene, xylene, 80% cyclohexane–20% cyclohexanol blend, and 40% cyclohexane–60% xylene blend. Toluene and xylene outperformed all the other solvents in the solubility tests of each method. The two blends chosen had the best results in the *RED* test, too. Then, these four solvents were used in the polymer dissolution equations to determine which one is the best for the process, since miscibility alone is not enough to optimise the complex process of dissolution.

### 3.2. Operating Procedure

The plastic feedstock needs to undergo pre-treatment before it can be mixed with the solvent and be dissolved. It has been found, experimentally, that waste plastic needs to be washed and shredded, which is something that would promote faster dissolution times due to smaller molecular weight and the absence of contaminants [4,14]. Moreover, the up-to-date model for plastic dissolution is to dissolve each polymer at a different temperature. However, this is usually done in processes where the plastics need to be obtained in their pure form [20]. In this design, the plastics do not need to be separated afterwards; the slurry of the solvent and plastic will be used as a carbon feedstock for various applications that could include the creation of carbon nanotubes [6,21] or graphene [22] via the chemical vapour deposition method.

The waste plastic feedstock containing PS, HDPE, PP, and ABS could either be brought from other plastic waste-sorting facilities or sorted in a plant from a mixture containing all of these kinds of waste plastic. The best choice would be to buy the separated waste from other facilities, since the required mixed plastic feedstock is just 1.2 tonnes per day, which is a very small percentage compared to the overall waste plastic treated in other plants. Moreover, until the model treats every kind of plastic apart from the four types selected (PS, HDPE, PP, ABS), the sorting process would be more time and energy consuming than the money to buy already sorted plastic. Therefore, in the proposed design, the plastics bought from an external vendor will be inspected and if not in the correct size, they will be shredded in a granulator and then fed to the dissolution vessel. The vessel will also be filled with the correct amount of solvent, and the temperature will be risen to the operating temperature, which is specified according to the solvent selected. Atmospheric pressure will be maintained throughout normal operation, and the residence time will be chosen according to the solvent selected. When the batch of plastics is dissolved, the slurry will be processed to a storage tank where it will be stored for further processing. 

### 3.3. Material Balance and Dissolution Times

#### 3.3.1. Assumptions

In order to perform mass and energy balances, several assumptions about the material selection, feedstock concentration, and polymer to solvent ratio had to be made. The number of repeated units for each polymer was assumed so that each polymer had a molecular weight that could be dissolved relatively quickly. Therefore, for N_PS_ = N_PP_ = N_ABS_ = 100 and N_PE_ = 200, PS had a molecular weight of 10,415 kg/kmol, HDPE had 5611 kg/kmol, PP had 4608 kg/kmol, and ABS had 21,130 kg/kmol respectively. The small molecular weight was used to account for the shredding of the plastics. Moreover, the density of all the materials used was assumed to be constant, and their values at 25 °C were used for simplicity. The same was applied for the viscosity of the solvents, where the viscosity at 25 °C was used and assumed to be constant. Regarding the viscosity of mixed solvents, a simple formula (Kendall and Monroe) was used to calculate the viscosity of the mixture [23].

(22) $$\eta_{m}{}^{\frac{1}{3}}=x_{1}\eta_{1}{}^{\frac{1}{3}}+x_{2}\eta_{2}{}^{\frac{1}{3}}\;\;\;$$

where *η_i_*, *x_i_*, are the solvent viscosity and molar fraction of each solvent, respectively, whereas *η_m_* is the viscosity of the solution. 

Regarding the choice of operating temperature, it would be 2.0 °C lower than the boiling point of the solvent. This would prevent gas formation as well as separation of the mixture of solvents by distillation.

Regarding the dissolution process, it was assumed that the solvent diffusion into the polymer was much faster than the disentanglement rate due to the polymers’ high molecular weight; therefore, the disentanglement process was the rate-limiting step. Furthermore, it was assumed that after 95% of the plastic was dissolved, the dissolution would be completed.

All the assumptions were used to calculate the reptation time as well as the disentanglement rate for each polymer. The mass fraction of polymer dissolved over time, *m_d_*, was assumed to vary linearly with time according to the following relationship.

(23) $$m_{d}=k_{d}t_{d}+t_{rep}\;\;$$

#### 3.3.2. Plastic Dissolution

The results for the reptation and disentanglement time of each polymer in the selected solvents are summarised in Table 6. The residence time was calculated as the sum of the maximum values of the reptation and disentanglement times of the polymer for each PSC.

The dissolution was proven to rely heavily on the disentanglement time. The reptation time of each polymer was insignificant compared to the disentanglement time, as the first occurred in a matter of seconds, whereas the latter needed a couple of hours to be completed. The polymer volume fraction, the temperature, the number of monomers in the polymer chain, and the solvent viscosity played an important role in the process, as also proven by Equation (21). However, the temperature of the process will always be approximately 300–400 K due to limitations regarding evaporation of the substances. The more important parameters were polymer volume fraction, number of monomers in the chain, and viscosity. These parameters are inversely proportional to the disentanglement rate. In addition, the polymer volume fraction and number of monomers in chain factors are raised to the power of 1.9 and 2.9, respectively, meaning that they have a larger effect on the process than the viscosity. However, the need to cut down plastics and thus reduce the number of monomers in the chain as well as the small percentage of plastic used in PSCs, which is below 20 wt %, have already been known from theoretical predictions and have already been performed in experimental procedures [14,20]. Therefore, cutting the plastics and mixing only a small amount with a large amount of solvent has been a standard operating procedure for years. The single most influential parameter for the disentanglement time was solvent and its related viscosity. 

Selecting a solvent with a low viscosity was very important for the process, as also seen by the results in Table 4. The solvents or solvent blends with higher viscosities tend to impede the disentanglement of the plastics. In this case, toluene had the lowest viscosity, about half the viscosity of the other blends; therefore, it had the fastest disentanglement rate out of all the others. The cyclohexane–cyclohexanol blend had the highest viscosity owing it to cyclohexanol, since alcohols tend to have higher viscosities than alkanes with the same number of carbons. The difference between the viscosity of pure xylene and the xylene–cyclohexane blend was minimal. A comparison between ketones and alcohols with the same number of carbons shows that ketones are less viscous, so a ketone could be used instead of an alcohol for solvent blends. Generally, it would be a good idea to find blends of organic solvents that can not only dissolve the required plastics but have very low viscosities, too.

However, some parameters were considered in neither the reptation time nor disentanglement rate equations. A change in pressure could not be taken into account according to Formulas (20) and (21). Therefore, the possibility to increase the temperature without boiling the mixture could not be exploited, as the increase in pressure could not be accounted for. Additionally, there was no relation between the solubility and the dissolution equations, which raised some concerns regarding the validity of the dissolution equations. For example, any liquid could be used in the dissolution equations regardless of its affinity with plastics or not. Therefore, the dissolution equations are prone to human error, because even a non-solvent could be used, and it would yield a sensible result by calculation, but not in practice. Therefore, future work will be carried out to further review the mathematical model based on empirical evidence. This will likely lead to a stronger axiomatic reasoning when the boundary conditions are more fully refined. For example, in the case of agitation, we determined that the agitation rate would have no effect on the process, as it was not in any of the formulas. However, there have been reports that agitation reduces the dissolution time by 10% [14].

Overall, toluene was proven to be the best solvent for the mixture of plastics as per the rapid timeframe required to complete the dissolution process, which is a little more than a day, compared to other solvents or mixtures, which take more than two days to completely dissolve the plastics. The dissolution time was observed to be almost equal to the disentanglement time, proving that the process is controlled by the polymer disentanglement. In the toluene PSC, PS and ABS were the fastest to be dissolved, whereas HDPE was significantly the slowest, making the dissolution of HDPE a rate-limiting factor of the process. 

**Figure 3:**
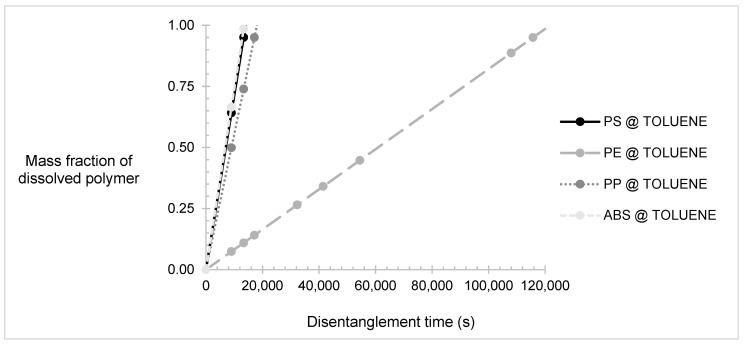
Mass fraction of polymers dissolved in toluene over time. Presenting polypropylene (PP), polystyrene (PS), high-density polyethylene (HDPE), and acrylonitrile–butadiene–rubber (ABS).

The mass fractions of plastics dissolved over time in toluene are presented in Figure 3. The reptation time was assumed to be negligible in relation to the dissolution time; therefore, Equation (23) was simplified to:

(24) $$m_{d}=k_{d}t_{d}.$$

A quantity of 28.75 m^3^·d^−1^ of toluene was needed to be mixed with 1.2 tonne·d^−1^ of plastic to achieve a flow rate of 1 tonne of slurry per hour. Since this would be a batch process, 24 tonnes would be used for each batch. The agitation rate of 300 rpm was an assumption adapted by other dissolution experiments [24]. This would help achieve faster dissolution by a matter of two hours. A batch time of 34 h was assumed considering filling, emptying the tank, and downtime to process the carbon feedstock. 

### 3.4. Notes on Applicability, Toluene Safety, Energy Requirements, and General Environmental Considerations

One should note that not all polymers will dissolve in toluene, as indicated by Figure 2, wherein S20 representing toluene (Table 5) is situated toward the bottom right corner of the Teas triangle; this should be interpreted to suggest that polymers far away from that location are less likely to be dissolved by toluene. Toluene is not a universal solvent, but it is a common solvent, to such a degree that the term “toluene dilution ratio” [25] is commonly used in industry.

Toluene is a known toxic substance and therefore is a regulated industrial chemical. However, it is a common industrial chemical; in the US alone, the annual production of toluene regularly surpasses 5 million metric tonnes [26]. It is typically used as a solvent for paints, lacquers, thinners, glues, correction fluid, and nail polish remover. It is commonly known to be a strong solvent and therefore is used as a cleaning agent, too. In all cases, toluene should be handled with the appropriate PPE should the work presented herein be attempted in person.

The process described herein considers the use of polymer–solvent slurry as the feedstock material for further processing, with the immediate example being the synthesis of carbon nanomaterials [21]; however, there may be advantages to reconstituting the polymer from the solvent instead [27]. This process of cleaning the polymer and solvent in a dissolution–reprocessing technique [28] would require additional operational units that incur greater energy demand. Moreover, this work has also used the assumption that solvents are in pristine condition and we have neglected energy requirements for cleaning/drying solvents in advance of creating PSCs. However, even though variations in solvent quality were not considered as a variable factor in this work, this should be considered in practical situations, because solvent quality can deviate from the ideal, which impacts their applicability in dissolution trials.

Given the challenging situation in which plastic waste is increasing in numbers [29], highly engaged industrial entities and policymakers [30] are faced with a simple question: what to do with the growing body of waste plastic materials? There are currently three common approaches to constructively tackle this issue; even though some may choose to obstruct or negate progress by continuing with the model of business as usual, there are others who are keenly aware of the growing opportunity in this sector and the growing need to prevent further geological spoil. The three common technical approaches are energy reclamation via combustion [31], mechanical recycling [32], and chemical recycling through pyrolysis [21,33,34]. A life cycle assessment comparing each route [35] suggests that each process (mechanical vs. chemical recycling) has different pollution levels and that chemical recycling is less polluting compared to present-day mechanical recycling. When compared against energy recovery from plastics, the use of chemical recycling with pyrolysis has a 50% lower climate change impact and has a smaller energy demand, too, thus pointing to the possibility of chemical recycling as a viable pathway to solve the growing plastic problem and create an open-loop pathway for materials manufacturing.

## 4. Conclusions

Open-loop recycling provides a more sustainable solution to the plastics pollution problem than other recycling techniques because the lifetime of the hydrocarbon is extended beyond the first use as a plastic. This can be achieved on a large scale, with multiple plastic stream by utilisation of the dissolution process. We have described how to accomplish the dissolution of four model polymers of industrial and global relevance such as PS, HDPE, PP, and ABS. These were examined by the mathematical modelling approach of Narasimhan and Peppas, and the solubility of each plastic was tested in different solvents by three different methods: the Hansen *RED* method, the Hildebrand method and the Flory–Huggins method. Interestingly, the mixing of two “mediocre” solvents such as cyclohexane and cyclohexanol enhanced their solubility effects and even showed better solubility than pure organic “good” solvents, as seen by the model of Hansen. Then, the solubility results were graphically represented using a Teas graph. Many of the non-solvents characteristically contained either oxygen or nitrogen atoms, and they were typical non-aromatic, polar compounds. Two pure solvents and two binary solvent blends were selected for study based on the dissolution calculations. The study of plastics dissolution by “Molecular theories in a continuum framework” provided the required mathematical expressions to describe the process. The total time required for the dissolution of the plastic mixture in toluene, calculated from the mathematical model, was 32 h. It was mathematically proven that higher polymer molecular weight results in longer dissolution times, so the polymer molecular weight is crucial. The same applied to polymer weight percentage; hence, a higher polymer content in the PSC would significantly slow the process. However, the single most important process parameter was solvent selection due to its relation to the viscosity of the final PSC. Highly viscous solvents could significantly retard the dissolution process, affecting the total batch time; low-viscosity solvent such as toluene outperformed the other solvents. Globally speaking, is it advantageous to adopt solvent blends with low viscosity and high affinity to the desired polymers. Despite the notable findings of this research, some inconsistencies were also encountered. The main dissolution equations could not account for changes in pressure or agitation. Moreover, no solubility parameter could be connected to the dissolution equations, making them prone to human error, since even a non-solvent could be used in the formula. Therefore, the equations should be empirically confirmed before building engineering operational units. Nonetheless, chemical recycling of plastics via the dissolution process was outlined and proven by mathematical expressions, which has been confirmed by previous works. Polymer dissolution is a complex process but accurate modelling combining empirical and theoretical methods lays the groundwork for higher industrial throughputs.

## 5. Patents

One patents have been filed from this work. A.O.W. filed: PROCESS FOR REUSE OF PLASTIC. United States Patent Application 20190375639 is owned by TRIMTABS Ltd. (Swansea, Wales, UK).

## Figures and Tables

**Table 1:** Affinity of plastic solubility in various solvents using the Hansen solubility method of Relative Energy Difference.

RED									Average	
Solvent/Polymer	PS	Affinity	PP	Affinity	HDPE	Affinity	ABS	Affinity	*RED*	Affinity
Acetone	1.8	Low	2.2	Low	3.3	Low	1.0	Low	2.1	Low
Acetonitrile	2.9	Low	3.2	Low	5.5	Low	2.0	Low	3.4	Low
Benzene	0.9	High	0.2	High	1.5	Low	0.9	High	0.9	High
Chloroform	0.6	High	0.9	High	1.6	Low	0.4	High	0.9	High
m-Cresol	1.9	Low	2.2	Low	3.7	Low	0.9	High	2.2	Low
Cyclohexanol	2.0	Low	2.2	Low	3.6	Low	0.9	High	2.2	Low
Cyclohexanone	0.6	High	1.3	Low	2.2	Low	0.7	High	1.2	Low
1,2 Dichlorobenzene	0.4	High	1.2	Low	2.6	Low	1.0	Low	1.3	Low
Dichloromethane	0.7	High	1.4	Low	2.4	Low	0.7	High	1.3	Low
Dimethylformamide	2.4	Low	2.9	Low	4.9	Low	1.5	Low	2.9	Low
Ethanol	3.4	Low	3.5	Low	5.8	Low	1.8	Low	3.6	Low
Ethyl acetate	1.3	Low	1.5	Low	2.0	Low	0.4	High	1.3	Low
Heptane	1.6	Low	0.9	High	1.0	Low	1.0	Low	1.1	Low
Hexadecane	1.3	Low	0.6	High	0.9	High	1.0	High	1.0	High
Hexafluoro-2-propanol	2.3	Low	2.4	Low	4.0	Low	1.0	Low	2.4	Low
Hexane	1.7	Low	1.0	Low	1.1	Low	1.0	Low	1.2	Low
Methanol	4.1	Low	4.2	Low	7.1	Low	2.3	Low	4.4	Low
Methyl Ethyl Ketone	1.3	Low	1.8	Low	2.7	Low	0.9	High	1.7	Low
Tetrahydrofuran	1.2	Low	1.6	Low	2.3	Low	0.4	High	1.4	Low
Toluene	0.6	High	0.3	High	1.3	Low	0.8	High	0.8	High
Xylene	0.7	High	0.4	High	1.0	Low	0.6	High	0.7	High
15% Acetonitrile 85% Toluene	0.4	High	0.7	High	1.4	Low	0.7	High	0.8	High
40% Cyclohexanone 60% Toluene	0.8	High	0.2	High	1.1	Low	0.8	High	0.7	High
50% Cyclohexanone 50% Xylene	0.4	High	1.0	High	1.7	Low	0.6	High	0.9	High
80% Cyclohexane 20% Cyclohexanol	0.9	High	0.5	High	0.6	High	0.6	High	0.7	High
87% Cyclohexane 13% Ethanol	2.2	Low	2.4	Low	3.8	Low	1.0	High	2.4	Low
25% Heptane 75% Xylene	0.9	High	0.4	High	0.7	High	0.7	High	0.7	High
25% Cyclohexane 75% Toluene	0.8	High	0.2	High	1.1	Low	0.8	High	0.7	High
40% Cyclohexane 60% Xylene	0.9	High	0.3	High	0.8	High	0.8	High	0.7	High

**Table 2:** Affinity of plastic solubility in various solvents using the Hildebrand solubility parameters.

Solvent	PS	Affinity	PP	Affinity	HDPE	Affinity	ABS	Affinity	Average	Affinity
Acetone	0.7	High	1.9	High	3.7	High	2.0	High	2.1	High
Acetonitrile	5.1	Low	6.4	Low	8.1	Low	6.4	Low	6.5	Low
Benzene	0.8	High	0.5	High	2.2	High	0.5	High	1.0	High
Chloroform	0.3	High	0.9	High	2.7	High	1.0	High	1.2	High
m-Cresol	3.5	High	4.7	Low	6.5	Low	4.7	Low	4.8	Low
Cyclohexanol	3.1	High	4.4	Low	6.1	Low	4.4	Low	4.5	Low
Cyclohexanone	0.3	High	1.5	High	3.3	High	1.6	High	1.7	High
1,2 Dichlorobenzene	1.2	High	2.4	High	4.2	Low	2.5	High	2.6	High
Dichloromethane	0.9	High	2.2	High	3.9	Low	2.2	High	2.3	High
Dimethylformamide	5.6	Low	6.8	Low	8.6	Low	6.9	Low	7.0	Low
Ethanol	7.3	Low	8.5	Low	10.3	Low	8.5	Low	8.6	Low
Ethyl acetate	1.1	High	0.1	High	1.9	High	0.2	High	0.8	High
Heptane	4.0	Low	2.7	High	1.0	High	2.7	High	2.6	High
Hexadecane	3.0	High	1.7	High	0.0	High	1.7	High	1.6	High
Hexafluoro-2-propanol	3.8	Low	5.0	Low	6.8	Low	5.1	Low	5.2	Low
Hexane	4.4	Low	3.1	High	1.4	High	3.1	High	3.0	High
Methanol	10.3	Low	11.6	Low	13.3	Low	11.6	Low	11.7	Low
Methyl ethyl ketone	0.2	High	1.0	High	2.8	High	1.1	High	1.3	High
Tetrahydrofuran	0.2	High	1.4	High	3.2	High	1.5	High	1.6	High
Toluene	1.1	High	0.1	High	1.9	High	0.2	High	0.8	High
Xylene	1.4	High	0.1	High	1.6	High	0.1	High	0.8	High

**Table 3:** Affinity of plastic solubility in various solvents using the Flory–Huggins method.

x_1,2_	PS	Affinity	PP	Affinity	HDPE	Affinity	ABS	Affinity	Average	Affinity
Acetone	0.7	Low	1.3	Low	0.8	Low	0.5	High	0.9	Low
Acetonitrile	2.4	Low	3.8	Low	3.1	Low	2.4	Low	3.1	Low
Benzene	0.2	High	0.0	High	0.2	High	0.5	High	0.1	High
Chloroform	0.1	High	0.3	High	0.2	High	0.1	High	0.2	High
m-Cresol	1.0	Low	1.7	Low	1.4	Low	0.5	Low	1.4	Low
Cyclohexanol	1.2	Low	1.8	Low	1.3	Low	0.5	High	1.4	Low
Cyclohexanone	0.1	High	0.6	Low	0.5	Low	0.3	High	0.4	High
1,2 Dichlorobenzene	0.1	High	0.5	Low	0.7	Low	0.6	Low	0.4	High
Dichloromethane	0.1	High	0.7	Low	0.6	Low	0.3	High	0.5	High
Dimethylformamide	1.6	Low	3.0	Low	2.5	Low	1.4	Low	2.4	Low
Ethanol	3.2	Low	4.4	Low	3.4	Low	1.9	Low	3.7	Low
Ethyl acetate	0.5	High	0.9	Low	0.4	High	0.1	High	0.6	Low
Heptane	0.7	Low	0.3	High	0.1	High	0.6	Low	0.4	High
Hexadecane	0.5	High	0.1	High	0.1	High	0.6	Low	0.2	High
Hexafluoro-2-propanol	1.5	Low	2.1	Low	1.6	Low	0.6	Low	1.7	Low
Hexane	1.1	Low	0.5	Low	0.2	High	0.9	Low	0.6	Low
Methanol	2.0	Low	2.6	Low	2.1	Low	1.4	Low	2.2	Low
Methyl ethyl ketone	0.5	High	1.0	Low	0.7	Low	0.4	High	0.7	Low
Tetrahydrofuran	0.4	High	0.9	Low	0.5	Low	0.1	High	0.6	Low
Toluene	0.1	High	0.0	High	0.2	High	0.4	High	0.1	High
Xylene	0.2	High	0.1	High	0.1	High	0.3	High	0.1	High

**Table 4:** List of polymer numbers relevant to Figure 2.

Polymer Number	Polymer
P1	Polystyrene
P2	Polypropylene
P3	High-density polyethylene
P4	Acrylonitrile–butadiene–styrene

**Table 5:** List of solvent numbers relevant to Figure 2.

Solvent Number	Solvent
S1	Acetone
S2	Acetonitrile
S3	Benzene
S4	Chloroform
S5	m-Cresol
S6	Cyclohexanol
S7	Cyclohexanone
S8	1,2 Dichlorobenzene
S9	Dichloromethane
S10	Dimethylformamide
S11	Ethanol
S12	Ethyl acetate
S13	Heptane
S14	Hexadecane
S15	Hexafluoro-2-propanol
S16	Hexane
S17	Methanol
S18	Methyl ethyl ketone
S19	Tetrahydrofuran
S20	Toluene
S21	Xylene

**Table 6:** Characteristic dissolution times of the plastic feedstock in selected solvent combinations.

Solvent	Viscosity	Solvent Volume Fraction	Polymer	Temperature	Polymer Volume Fraction	Reptation Time	Disentanglement Rate	Disentanglement Time
	*η*	*u*_1_		*T*	*u*_2_	*t_rep_*	*k_d_*	*t_d_*
	Pas			°C		s	s^−1^	h
Toluene	2.69 × 10^−4^	9.60 × 10^−1^	PS	108	1.00 × 10^−2^	8.83 × 10^−6^	7.12 × 10^−5^	4
			HDPE		1.00 × 10^−2^	1.08 × 10^−4^	8.21 × 10^−6^	32
			PP		1.10 × 10^−2^	1.14 × 10^−5^	5.54 × 10^−5^	5
			ABS		0.90 × 10^−2^	8.52 × 10^−6^	7.38 × 10^−5^	4
Xylene	6.03 × 10^−4^	9.57 × 10^−1^	PS	137	1.00 × 10^−2^	2.14 × 10^−5^	2.94 × 10^−5^	9
			HDPE		1.10 × 10^−2^	2.62 × 10^−4^	3.39 × 10^−6^	78
			PP		1.20 × 10^−2^	2.75 × 10^−5^	2.29 × 10^−5^	12
			ABS		1.00 × 10^−2^	2.06 × 10^−5^	3.05 × 10^−5^	9
80% Cyclohexane 20% Cyclohexanol	7.58 × 10^−4^	9.59 × 10^−1^	PS	78	1.00 × 10^−2^	2.80 × 10^−5^	2.24 × 10^−5^	12
			HDPE		1.10 × 10^−2^	3.44 × 10^−4^	2.59 × 10^−6^	102
			PP		1.10 × 10^−2^	3.60 × 10^−5^	1.75 × 10^−5^	15
			ABS		1.00 × 10^−2^	2.71 × 10^−5^	2.33 × 10^−5^	12
40% Cyclohexane 60% Xylene	5.96 × 10^−4^	9.58 × 10^−1^	PS	78	1.00 × 10^−2^	2.29 × 10^−5^	2.75 × 10^−5^	9
			HDPE		1.10 × 10^−2^	2.81 × 10^−4^	3.17 × 10^−6^	83
			PP		1.10 × 10^−2^	2.94 × 10^−5^	2.14 × 10^−5^	12
			ABS		1.00 × 10^−2^	2.21 × 10^−5^	2.85 × 10^−5^	12

## Supplementary Materials

The following are available online at https://www.mdpi.com/article/10.3390/polym14010112/s1, Table S1: Hansen and Hildebrand solubility parameters of polymers and solvents, Table S2: Solvents’ distance from the centre for the *RED* method, Table S3: Flory–Huggins parameters of selected solvents, Table S4: Fractional parameters of plastics and polymers for the creation of the Teas graph.
