# Supplementary material for: The Mechanics of Forming Ideal Polymer–Solvent Combinations for Open-Loop Chemical Recycling of Solvents and Plastics
Source: Polymers (Basel). 2021 Dec 29;14(1):112. doi: 10.3390/polym14010112 (PMC8747207; doi:10.3390/polym14010112)
Supplement: Supplementary file 1 [file polymers-14-00112-s001.zip › The Mechanics of Forming Ideal Polymer¿CSolvent Combinations for Open-Loop Chemical Recycling of Solvents and Plastics.pdf]

Supplementary file

The Mechanics of Forming Ideal Polymer–Solvent Combinations for Open-Loop Chemical Recycling of Solvents and Plastics

Ioannis Tsampanakis[1,2], Alvin Orbaek White[1,2] *

[1] Energy Safety Research Institute, Swansea University, Bay Campus, Swansea SA1 8EN, UK; alvin.orbaekwhite@swansea.ac.uk (A.O.W.); 958003@Swansea.ac.uk (I.T.)

[2] Chemical Engineering, Faculty of Science and Engineering, Swansea University, Bay Campus, Swansea, SA1 8EN UK; alvin.orbaekwhite@swansea.ac.uk (A.O.W.); 958003@Swansea.ac.uk (I.T.)

* Correspondence: alvin.orbaekwhite@swansea.ac.uk (A.O.W.)

Table S1. Hansen & Hildebrand solubility parameters of polymers & solvents

Material	δ_D Mpa$^{0.5}$	δ_P Mpa$^{0.5}$	δ_H Mpa$^{0.5}$	δ_1 Mpa$^{0.5}$	Interaction radius R_0
**Polymers**					
Polystyrene (PS)	18.5	4.5	2.9	19.3	5.3
Polypropylene (PP)	18	0	1	18.0	6
Polyethylene - High Density (HDPE)	16	0.8	2.8	16.3	3.2
Acrylonitrile - Butadiene - Styrene (ABS)	16.3	2.7	7.1	17.98	7.8
**Solvents**					
Acetone	15.5	10.4	7	19.9	74
Acetonitrile	15.3	18	6.1	24.4	52.6
Benzene	18.4	0	2	18.5	89.4
Chloroform	17.8	3.1	5.7	18.9	80.7
m-Cresol	18	5.1	12.9	22.7	104.7
Cyclohexanol	17.4	4.1	13.5	22.4	106
Cyclohexanone	17.8	6.3	5.1	19.6	104
1,2 Dichlorobenze	19.2	6.3	3.3	20.5	112.8
Dichloromethane	18.2	6.3	6.1	20.2	N/A
Dimethylformamide	17.4	13.7	11.3	24.9	77
Ethanol	15.8	8.8	19.4	26.5	58.5
Ethyl acetate	15.8	5.3	7.2	18.2	98.5
Heptane	15.3	0	0	15.3	147.4
Hexadecane	16.3	0	0	16.3	294.1
Hexafluoro-2-propanol	17.2	4.5	14.7	23.1	105.3
Hexane	14.9	0	0	14.9	131.6
Methanol	15.1	12.3	22.3	29.6	40.7
Methyl Ethyl Ketone	16	9	5.1	19.1	90.1
Tetrahydrofuran	16.8	5.7	8	19.5	81.7
Toluene	18	1.4	2	18.2	106.8
Xylene	17.6	1	3.1	17.9	123.3

Table S2. Solvents' distance from the centre for the RED method

Distance from the Centre				
R_a	PS	PP	HDPE	ABS
Acetone	9.4	13.0	10.5	7.9
Acetonitrile	15.3	19.5	17.6	15.5
Benzene	4.6	1.3	4.9	7.1
Chloroform	3.4	5.6	5.2	3.3
m-Cresol	10.1	12.9	11.7	7.1
Cyclohexanol	10.8	13.2	11.5	6.9
Cyclohexanone	3.2	7.5	7.0	5.1
1,2 Dichlorobenze	2.3	7.1	8.5	7.8
Dichloromethane	3.7	8.1	7.8	5.3
Dimethylformamide	12.7	17.2	15.7	12.0
Ethanol	17.9	20.9	18.4	13.8
Ethyl acetate	6.9	9.3	6.3	2.8
Heptane	8.3	5.5	3.2	7.9
Hexadecane	6.9	3.5	3.0	7.6
Hexafluoro-2-propanol	12.1	14.5	12.7	8.0
Hexane	9.0	6.3	3.6	8.1
Methanol	22.0	25.3	22.7	18.1
Methyl Ethyl Ketone	7.1	10.7	8.5	6.6
Tetrahydrofuran	6.2	9.3	7.3	3.3
Toluene	3.4	1.7	4.1	6.3
Xylene	3.9	2.5	3.2	5.1
15% Acetonitrile<br>85% Toluene	1.9	4.3	4.4	5.3
40% Cyclohexanone<br>60% Toluene	4.5	1.3	3.4	6.6
50% Cyclohexanone<br>50% Xylene	2	5.7	5.3	4.6
80% Cyclohexane<br>20% Cyclohexanol	4.9	3	1.8	4.8
87% Cyclohexanol<br>13% Ethanol	11.7	14.2	12.3	7.7
25% Heptane<br>75% Xylene	4.8	2.5	2.1	5.4
25% Cyclohexane<br>75% Toluene	4	1.3	3.6	6.4
40% Cyclohexane<br>60% Xylene	4.7	1.8	2.7	5.9

Table S3. Flory-Huggins parameters of selected solvents

Solvent	V(cm^3/mol)	$A_{1,2}$			
		PS	PP	HDPE	ABS
Acetone	74	21.9	42.3	27.7	15.5
Acetonitrile	100	58.4	94.8	77.2	59.8
Benzene	89.4	5.3	0.4	6.1	12.7
Chloroform	80.7	2.9	8.0	6.7	2.8
m-Cresol	100	25.3	41.9	34.1	12.7
Cyclohexanol	100	29.3	43.6	33.3	11.9
Cyclohexanone	104	2.5	14.2	12.1	6.5
1,2 Dichlorobenze	100	1.3	12.7	17.9	15.3
Dichloromethane	100	3.5	16.5	15.1	7.1
Dimethylformamide	100	40.0	73.8	61.6	35.9
Ethanol	100	80.0	108.8	84.9	47.4
Ethyl Acetate	98.5	12.1	21.5	9.9	1.9
Heptane	100	17.4	7.5	2.6	15.4
Hexadecane	100	12.0	3.1	2.2	14.4
Hexafluoro-2-propanol	100	36.5	52.6	40.3	16.1
Hexane	131.6	20.1	9.9	3.3	16.4
Methanol	40.7	120.9	159.7	128.9	82.2
Methyl Ethyl Ketone	90.1	12.5	28.5	18.1	11.0
Tetrahydrofuran	100	9.8	21.8	13.4	2.7
Toluene	106.8	2.9	0.7	4.3	9.8
Xylene	123.3	3.9	1.5	2.6	6.4

Table S4. Fractional parameters of plastics & polymers for the creation of the Teas graph

**Polymers / Solvents**	**F_D**	**F_P**	**F_H**
Polystyrene (P1)	71.4	17.4	11.2
Polypropylene (P2)	94.7	0.0	5.3
Polyethylene - High Density (P3)	81.6	4.1	14.3
Acrylonitrile - Butadiene - Styrene (P4)	62.5	10.3	27.2
Acetone (S1)	47.1	31.6	21.3
Acetonitrile (S2)	38.8	45.7	15.5
Benzene (S3)	90.2	0.0	9.8
Chloroform (S4)	66.9	11.7	21.4
m-Cresol (S5)	50.0	14.2	35.8
Cyclohexanol (S6)	49.7	11.7	38.6
Cyclohexanone (S7)	61.0	21.6	17.5
1,2 Dichlorobenzene (S8)	66.7	21.9	11.5
Dichloromethane (S9)	59.5	20.6	19.9
Dimethylformamide (S10)	41.0	32.3	26.7
Ethanol (S11)	35.9	20.0	44.1
Ethyl acetate (S12	55.8	18.7	25.4
Heptane (S13)	100.0	0.0	0.0
Hexadecane (S14)	100.0	0.0	0.0
Hexafluoro-2-propanol (S15)	47.3	12.4	40.4
Hexane (S16)	100.0	0.0	0.0
Methanol (S17)	30.4	24.7	44.9
Methyl Ethyl Ketone (S18)	53.2	29.9	16.9
Tetrahydrofuran (S19)	55.1	18.7	26.2
Toluene (S20)	84.1	6.5	9.3
Xylene (S21)	81.1	4.6	14.3
